# Effects of a 12-Week Interventional Exercise Programme on Muscle Strength, Mobility and Fitness in Patients With Diabetic Foot in Remission: Results From BIONEDIAN Randomised Controlled Trial

**DOI:** 10.3389/fendo.2022.869128

**Published:** 2022-07-05

**Authors:** Eliška Vrátná, Jitka Husáková, Radka Jarošíková, Michal Dubský, Veronika Wosková, Robert Bém, Alexandra Jirkovská, Kateřina Králová, Bára Pyšková, Věra Lánská, Vladimíra Fejfarová

**Affiliations:** ^1^ Faculty of Physical Education and Sport, Charles University, Prague, Czechia; ^2^ Clinical Rehabilitation Division, Institute for Clinical and Experimental Medicine, Prague, Czechia; ^3^ Diabetes Centre, Institute for Clinical and Experimental Medicine, Prague, Czechia; ^4^ Second Faculty of Medicine, Charles University, Prague, Czechia

**Keywords:** diabetic foot, physical activity, diabetes mellitus, exercise, education

## Abstract

**Objectives:**

Diabetic foot syndrome (DFS) is a serious late diabetic complication characterised by limited joint mobility and other biomechanical and muscle abnormalities.

**Aim:**

To evaluate the effect of an interventional exercise programme on anthropometric parameters, muscle strength, mobility and fitness in patients with diabetic foot in remission.

**Data Sources and Study Selection:**

Thirty-eight patients with type 2 diabetes and DFS without active lesions (mean age 65 ± 6.9 years, BMI 32 ± 4.7 kg.m^-2^, waist-hip ratio (WHR)1.02 ± 0.06) were enrolled in our randomised controlled trial. All subjects were randomised into two groups: an intervention group (I; n=19) and a control group (C; n=19). The 12-week exercise intervention focused on ankle and small-joint mobility in the foot, strengthening and stretching of the lower extremity muscles, and improvements *in* fitness. Changes (Δ=final minus initial results) in physical activity were assessed using the International Physical Activity Questionnaire (IPAQ), with joint mobility detected by goniometry, muscle strength by dynamometry, and fitness using the Senior *Fitness* Test (SFT).

**Data extraction:**

Due to reulceration, 15.8% of patients from group I (3/19) and 15.8% of patients from group C were excluded. Based on the IPAQ, group I was more active when it came to heavy (p=0.03) and moderate physical activity (p=0.06) after intervention compared to group C. Group I improved significantly in larger-joint flexibility (p=0.012) compared to controls. In group I, dynamometric parameters increased significantly in both lower limbs (left leg; p=0.013, right leg; p=0.043) compared to group C. We observed a positive trend in the improvement of fitness in group I compared to group C. We also confirmed positive correlations between heavy physical activity and selected parameters of flexibility (r=0.47; p=0.007), SFT (r=0.453; p=0.011) and dynamometry (r=0.58; p<0.0025). Anthropometric parameters, such as BMI and WHR, were not significantly influenced by the intervention programme.

**Conclusion:**

Our 12-week interventional exercise programme proved relatively safe, resulting in improved body flexibility and increased muscle strength in DF patients in remission.

## Introduction

It is widely acknowledged that decreased muscle strength and mobility in the foot joints along with reduced walking speed are among the risk factors for diabetic foot ulcers (DFU), diabetic foot syndrome (DFS) and their recurrence (1–3). DFS occurs in 15-25% of patients with diabetes mellitus (DM). DFUs contribute to lower limb amputations in nearly 84% of all diabetic patients with DFS (4).

The main risk factor of DFS is diabetic sensorimotor neuropathy (5), a complication that contributes to the development of limited joint mobility (LJM) (1, 6). Along with atrophy of the leg and foot muscles, LJM results in biomechanical changes and increased plantar pressure in some regions. Overloading in certain parts of the foot leads to the formation of hyperkeratoses and subsequent DFUs (7, 8). Additionally, muscle atrophy and inactivity decreases overall patient mobility due to offloading during active DFS therapy, which frequently results in reduced patient fitness and disability.

Early prevention, which consists of diabetes control and inducing biomechanical changes in static (detected while standing) (9) and dynamic characteristics (gait abnormalities) (10), can reduce the risk of DFS, DFU and their recurrence. This could be achieved by controlled, structured physical activity (PA). Therefore, the aim of our study was to evaluate the effect of structured interventional exercise on muscle strength, mobility and fitness in patients with DFS in remission and also to verify the safety of the programme.

## Methods

### Study Subjects

A total of 38 patients with type 2 diabetes mellitus (DM) and DFS in remission (positive history of DFS, without active lesions; mean age 65 ± 6.9 years, HbA1c according to IFCC 59.2 ± 15.1 mmol/mol and BMI 32 ± 4.7 kg.m^-2^, WHR 1.01 ± 0.06) were enrolled in the BIONEDIAN randomised controlled trial. Individuals were monitored at our Outpatient Foot Clinic. Patients aged 30-70 years without active DFU or Charcot foot were included in the study. Exclusion criteria were: neuropathy of other aetiology, inability to exercise regularly, visual disturbance or its severe impairment, non-compliance, critical limb-threatening ischaemia, active DFU, surgical foot wound, active Charcot foot, active tumour, recent stroke, myocardial infarction (within 8 weeks prior to enrolment), recent percutaneous transluminal angioplasty, percutaneous coronary angioplasty or bypass (within 8 weeks prior to enrolment), myopathy, rheumatoid arthritis, coxarthrosis or gonarthrosis grade 3 – 4.

All participants were randomised into two groups based on stratified randomization to balance the influence of age, diabetes duration, HbA1c and ischemia (TcPO2): an intervention group (group I) and a control group (group C). Each group consisted of 19 patients. Examinations were performed by a podiatrist and a physiotherapist at the beginning of the study and at the end of the study (12 weeks after enrolment) and whenever there were changes in clinical/foot status. Prior to inclusion in the study, signed informed consent was obtained from all patients and approved by FTN and IKEM’s ethics committee.

### Assessment of Physical Activity, Muscular and Biomechanical Changes

#### Physical Activity Assessment

The International Physical Activity Questionnaire (IPAQ) is a self-reported measure used to obtain data on the total amount and intensity of physical activity (PA) performed in one week. The questionnaire comes in short and long versions. The short version, which was used for this research, measures the distribution of walking time as well as moderately intense and intense PA over the preceding 7 days. PA levels are assessed based on the number of metabolic equivalent of task (MET = the objective measure of oxygen consumed at rest) minutes/week and further categorised as low, moderate or high based on movement recommendations (11–13). A high PA level means that the individual engaged in (i) high-intensity activity for at least 3 days, reaching 1500 MET minutes/week, or (ii) any combination of walking, medium-intensity or high-intensity activity on 7 or more days, totalling at least 3000 MET minutes/week. Moderate physical activity was defined as (i) 3 or more days of intense activity (at least 20 minutes per day) or (ii) 5 or more days of moderate or walking activity (at least 30 minutes per day) or (iii) 5 or more days of any combination of moderate-intensity walking or high-intensity activity. Participants meeting the above PA criteria were required to reach at least 600 MET minutes/week. Low levels of PA applied to any participant that failed to meet the criteria for moderate and high PA levels (<600 MET minutes/week). These patients were considered either only slightly active or physically inactive.

The IPAQ protocol was completed during the inclusion and final visit. Therefore, the physical activity of 7 day period before and at the end of study period were included into the IPAQ protocol. Therefore, the IPAQ completed at the end of the study period included data related to recommended exercise. We took IPAQ as an auxiliary test to check the patient adherence to intervention program.

The number of MET minutes/week and the number of minutes of high-, moderate- and low-intensity PA/week recorded during the intervention were compared between study groups.

#### Senior Fitness Test

The physical fitness of probands was assessed using the Senior Fitness Test (SFT) (14, 15), a battery of tests intended primarily for adults over the age of 60 (16). The test requires no special equipment and special space and can be performed anywhere. In order to minimise time and space requirements, we omitted the bicep raise and up-and-go exercises from the test battery. Instead of the 6-minute walk test, we used the 2-minute step-in-place test. This modified SFT involved four functional tests focusing on strength, strength, aerobic capacity, agility, coordination and speed:

1. *Sit and stand up Chair test*: From a seated position (43-cm-high chair without arm rests), the subject stands fully up and then sits fully back down again, repeating the action for 30 seconds. The aim is to perform as many repetitions as possible. The test assesses strength in the lower part of the body.

2. *The 2-minutes Step test in place*: Remaining in one place, the subject lifts the knees to reach a point similar the top of the hip bone. The total number of steps is counted for two minutes. The test is a measure of endurance (aerobic capacity). As was mentioned in the text, the traditional SFT examines endurance (aerobic capacity) use the 6-minute walk test. In our study, the test was replaced by a 2-minute step test (TST). The SFT manual offers the possibility to switch the tests in this way. The reason is to facilitate the performance of the test. The 2-minute step test does not require a large space for performance and long-term examination, which many patients with comorbidities may not easily undergo (52). The suitability of test switching is examined and confirmed by many studies (53-55).

3. *Sit and reach test*: Sitting on the edge of a chair, the foot of one leg is positioned flat on the floor and the other leg extended. Keeping the knee straight and heel on the floor, the subject reaches forward to touch the toes of the outstretched leg. The distance in centimetres between the fingertips and toes is then measured. The test assesses flexibility in the lower part of the body.

4. *Back scratch test*: In a standing position, one hand is placed over the shoulder behind the head and back, reaching as far as possible down the middle of the back. The other arm is placed behind the back with the palm of the hand facing outward, extending upward as far as possible. The distance between the tips of the middle fingers is then measured in centimetres. The test assesses flexibility in the upper part of the body.

Normal values for all SFT examinations across age categories are given in **Tables 1**, **2** (11, 13–17). After completing all four exercises from the SFT, the total sum of points from individual disciplines was calculated. The test was performed before and after the exercise intervention and the differences in total sums of points showed the effect of the structured exercise intervention program.

**Table 1:** SFT values – physiological ranges for women.

Ages	60 - 64	65 - 69	70 - 74	75 - 79	80 - 84	85 - 89
Sit and stand up Chair test (repetitions)	12 - 17	11 - 16	10 - 15	9 - 14	8 - 13	4 - 11
2-minutes step test in place (repetitions)	75 - 107	73 - 107	68 - 101	68 - 100	60 - 91	55 - 85
Sit and reach test (cm +/-)	-1.3 - + 12.7	-1.3 - +11.4	-2.5 - +10.2	-3.8 - +8.9	-5.1 - +7.6	-6.4 - +6.4
Back scratch test (cm +/-)	-7.6 ± 3.8	-8.9 - +3.8	-10.2 - +2.5	-12.7 - +1.3	-14 - +0.0	-14.5 - -2.5

**Table 2:** SFT values – physiological ranges for men.

Ages	60 - 64	65 - 69	70 - 74	75 - 79	80 - 84	85 - 89
Sit and stand up Chair test (repetitions)	14 - 19	12 - 18	12 - 17	11 - 17	10 - 15	8 - 14
2-minutes step test in place (repetitions)	87 - 115	86 - 116	80 - 110	73 - 109	71 - 103	59 - 91
Sit and reach test (cm +/-)	-6.4 - +10.2	-7.6 - +7.6	-8.9 - +6.4	-10.2 - +5.1	-14 - +3.8	-14 - +1.3
Back scratch (cm +/-)	-16.5 - +0	-19.1 - -2.5	-20.3 - -2.5	-22.7 - -5.1	-24.1 - -5.1	-25.4 - -7.6

SFT, Senior Fitness Test.

#### Muscle Strength *Detection*

**Figure 1:**
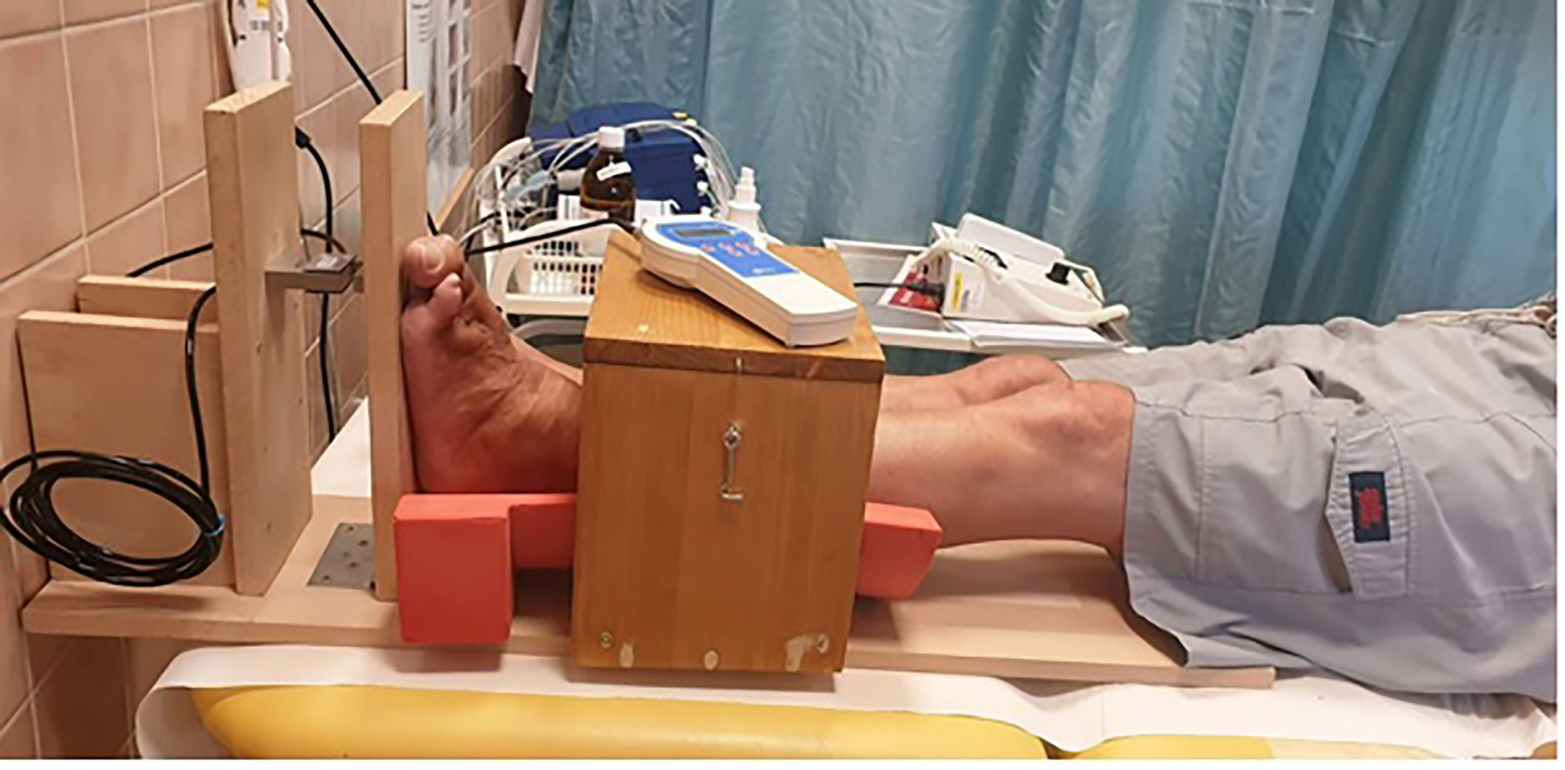
Dynamometry examination of maximal isometric plantar flexion muscle strength.

Another parameter evaluated as part of this interventional study was the force of isometric plantar flexion, determined using an instrument constructed at IKEM’s Magnetic Resonance Department (**Figure 1**) as previously described (18). Primarily used to measure the strength of different muscle groups, the device adjusts resistance during plantar flexion (knee flexion up to 60°) in the musculus gastrocnemius and musculus soleus (19, 20). When resistance is applied in the opposite direction, the device can measure strength in the musculus tibialis anterior (21). In this study, a compression sensor was used to measure maximal muscle force, defined as the highest value in Newtons achieved over 3 repetitions.

With instructions provided by the examiner, the test was performed as follows: In a supine position, the subject grips the sides of the bed with the hands while pushing with the legs against the device with as much force as possible. The measurement ends when the number of Newtons on the digital display stops rising. The test is repeated three times, with the highest value used for statistical analysis.

### A Structured Exercise Intervention Programme for Patients With DFS in Remission

The purpose of our structured intervention programme was to improve joint mobility and postural stability while increasing physical fitness in patients with DFS. Drawing from previous exercises (22, 23), our intervention focused on mobilisation, stabilisation and toning exercises in alternation with cyclic aerobic activity (walking). Participants were required to perform a minimum of 150 minutes of PA each week for 12 weeks.

The intervention exercises (designed to be performed at home) were demonstrated by a physiotherapist followed by podiatric consultations at an outpatient foot clinic as required. Participants received a detailed description of each exercise (training #1 and training #2; **Tables 3**, **4**) along with a training diary. Of the 11 exercises in the programme, 7 were designed to increase mobility, relax the muscles and tendons, strengthen muscles around the ankle, and improve foot proprioception. *All training was performed with moderate intensity (3 to 6 METs).*

**Table 3:** Training #1 performed 4 times per week.

		Instruction	Repetitions
1	Joint mobilisation – sole of foot	While seated, grasp your foot in your hands and squeeze, then stretch and relax the individual bones.	2 minutes (approx.) each foot
2	Alternating dorsal and plantar flexion in supine position (active exercise)	Lying on your back, point your toes towards your knee and again in the opposite direction as far as you can.	30 times
3	Circumduction in the ankle joint in supine position	Lying on your back, make 30 circles to the left and 30 circles to the right to the maximum extent as many times as you can with both feet.	30 times each foot
4	Dorsal and plantar ankle flexion while seated	While seated, lift your heels off the mat and then do the same with your toes to the maximum extent.	30 times
5	Toe flexor stretching	While seated, place your feet on the mat and stretch (raise) your toes as much as possible, then count to 5.	10 times
6	Strengthening of triceps surae (extensions)	Standing against the wall (ensuring stability), raise your heels off the floor and slowly return to a flat position.	30 times (3 x 10 reps)
7	Stability training – standing on one leg	Standing on one leg, step forward as far as you can with the other leg and then immediately backward in the opposite direction = 1 rep.	3 times, 10 reps for each leg
8	Proprioception	While seated, lift up a crumpled piece of paper lying on the floor.	30 seconds each foot, 3 times
9	ACT – standing up from a seated position	Sitting on a chair, try to stand up by pushing into the ground with your feet while pushing with your palms against the chair supports.	3 times, 10 reps
10	ACT – muscle activation in supine position with legs bent	Lie on your back with your legs bent at 90° and heels flat on the floor, then push down with your feet while applying pressure on your thighs with your hands. Hold for 3 seconds and then release for 3 seconds = 1 rep.	3 times, 10 reps
11	Stretching of m. triceps surae	Stand with one foot in front and the other foot behind, making sure you keep the heel of the back foot in contact with the ground. Count to 50.	Each leg twice

**Table 4:** Training #2 performed 3 times per week.

1	Joint mobilisation – sole of foot	While seated, grasp your foot in your hands and squeeze, then stretch and relax the individual bones.	2 minutes (approx.) each foot
2	Walking	Mild intensity, light breathing only.	30 minutes

**Figure 2:**
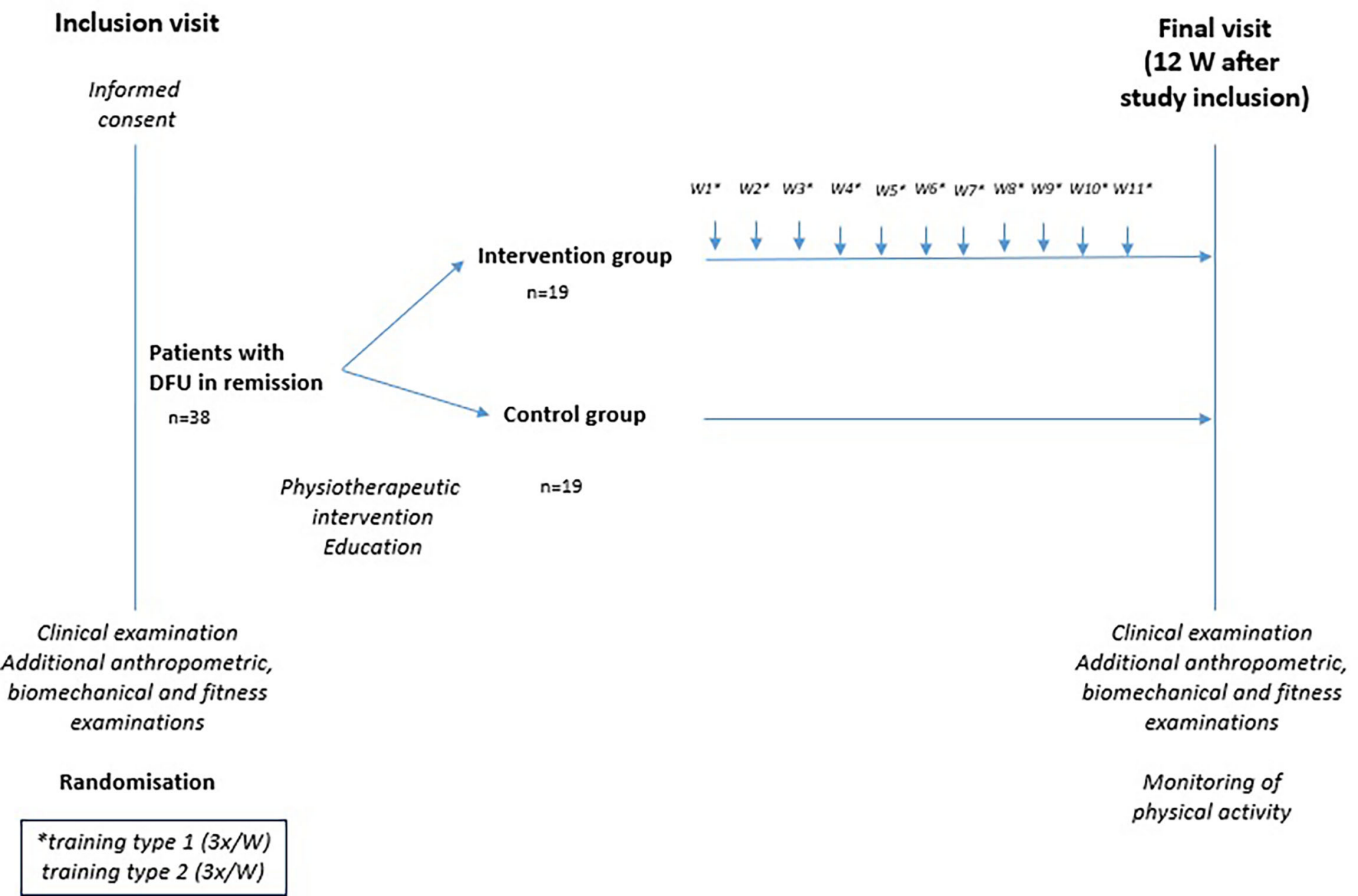
Study schedule.

The participants completed the interventional exercise programme over a 12-week period. Training #1 (**Table 3**) was performed 4 times a week and training #2 (**Table 4**) three times a week for a minimum of 30 minutes using appropriate footwear. Regular foot inspections were also carried out (for the full study schedule, see **Figure 2**). Participants were asked to record any additional PA performed during this period in a training diary. They were also instructed to inspect the lower limbs regularly throughout the intervention, to wear appropriate preventive footwear, and to contact the study physician immediately if any unusual changes occurred. The adherence and motivation of individuals were mainly determined by their subjective feeling.

The control group did not receive any recommendations on physical activity. In the study, they were only tested and completed questionnaires about their daily physical activity.

### Evaluation of the Structured Exercise Intervention Programme

Individuals were assessed at the Outpatient Foot Clinic of IKEM’s Diabetes Centre. During the initial visit, the patient underwent a podiatric examination carried out by a diabetologist. Medical history was also reviewed, with basic laboratory tests conducted to evaluate fasting glycaemia, glycated haemoglobin and serum creatinine levels and feet control. Participants were also seen by a physiotherapist to assess flexibility (see SFT), muscle strength (dynamometry of plantar flexion) and fitness (see SFT). Testing was followed by thorough descriptions of how to perform the exercise programme, details of the handover and instructions for the training diary. Patients returned 3 months later for a retest, which followed exactly the same format as the initial visit. The assessment was maximally blinded, each patient was evaluated anonymously only under a study number, final statistical processing was performed collectively after the end of study(follow-up). Once testing was complete, patients were interviewed to obtain a subjective evaluation of the exercise programme. Patients were informed that they were free to contact any of the physiotherapists as needed after completion of the study.

### Statistical Analysis

The Shapiro-Wilk test was used to test Gaussian distribution, with the t-test applied for Gaussian variables. For variables differing from Gaussian distribution, the Mann-Whitney test was used. Fisher’s exact test was used for discrete variables. To measure relations between variables, the correlation coefficient was used. A two-sided p-value less than.05 was considered statistically significant. All calculations were carried out using JMP^®^ 15.2.0 statistical software (2019 SAS Institute, Cary, *USA).*

Using statistical data on joint flexibility the required power of the study should be achieved upon enrolment of at least 24 subjects in each study arm. The physically active arm of the study was compared with the non-active arm based on standard observation and monitoring of patients. The Mann-Whitney U test was performed at a 0.05 two-sided significance level and at a power of 80%. However due to covid pandemic we had to finalize our study prematurely before inclusion of target number of study subjects.

## Results

Intervened patients did not differ significantly from controls in respect of basal characteristics, including anthropometric data such as BMI and WHR (**Table 5**). Only a non-significant slight reduction in hip circumference was detected post-intervention in group I compared to group C (p=0.19). Reulcerations leading to exclusion from the trial occurred during the study period with the same frequency in both groups (in 15.8% of individuals - 3/19).

**Table 5:** Comparison of basal characteristics between study groups.

	Intervened subjects (group I) n = 16	Control group (group C) n = 16	P-value
Age (years)	63.7 ± 7.3	66.1 ± 6.5	NS
Duration of DM (years)	15.1 ± 9.0	13.8 ± 7.4	NS
BMI (kg.m^-2^)	31.25 ± 5.2	32.5 ± 4.5	NS
WHR	1.02 ± 0.08	1.01 ± 0.04	NS
HbA1c (mmol/mol)	57.4 ± 13.3	59.5 ± 15.1	NS
Fasting glycaemia (mmol/l)	9.5 ± 3.8	10.2 ± 5.2	NS
TcPO2 – RLL (mmHg)	55.6 ± 8.5	49.4 ± 13.8	NS
TcPO2 – LLL (mmHg)	49.9 ± 13.2	53.4 ± 14.8	NS

n, number; BMI, body mass index; WHR, waist-hip ratio; HbA1c, glycosylated haemoglobin; TcPO2, transcutaneous oxygen pressure; mmHg, millimetres of mercury; RLL, right lower limb; LLL, left lower limb.

Based on the IPAQ questionnaire, group I was significantly more active. Both high- (p=0.03) and moderate-intensity (p=0.06) PA was greater compared to group C, a finding confirmed by training diary entries. Twelve participants advanced to high-intensity PA, with low-intensity PA reducing to a minimum *(p=0.031).* In the control group*(p=0.031)*, IPAQ results were almost identical to those obtained at the beginning of the study (**Table 6**).

**Table 6:** Comparison of anthropometric, SFT and dynamometry parameters as well as changes in PA between study groups.

Evaluated parameters		Intervened subjects (group I) n = 16	Control group (group C) n = 16	P-value
Weight (kg)	Pre-test	100.4 ± 19.2	100 ± 18.8	NS
	Post-test	100.1 ± 11.6	100.1 ± 11.4	NS
BMI (kg.m^-2^)	Pre-test	31.25 ± 5.2	32.46 ± 4.5	NS
	Post-test	31.2 ± 5.2	32.48 ± 4.5	NS
Waist circumference (cm)	Pre-test	114.25 ± 14.3	115.43 ± 11.4	NS
	Post-test	113.7 ± 13.8	114.8 ± 12.2	NS
		-0.6 ± 2.3	-0,6 ± 3.5	NS
Hip circumference (cm)	Pre-test	112.2 ± 8.9	114.4 ± 12.7	NS
	Post-test	111.8 ± 9	114.9 ± 12.8	NS
	Δ	-0.4 ± 2.2	0.5 ± 1.6	0.19
WHR	Pre-test	1.02 ± 0.08	1.01 ± 0.04	NS
	Post-test	1.02 ± 0.08	1 ± 0.05	NS
	Δ	-0.5	0	NS
Left shoulder flexibility	Pre-test	(-23.8) ± 10.1	(-23.7) ± 11.3	NS
	Post-test	(-22.1) ± 9.1	(-26.3) ± 12.0	NS
	Δ	1.6 ± 3.8	(-2.6) ± 5.0	0.012
Right shoulder flexibility	Pre-test	(-20.8) ± 13.4	(-22.6) ± 11.4	NS
	Post-test	(-19.6) ± 13.4	(-22.9) ± 13.5	NS
	Δ	1.1 ± 3.6	(-0.3) ± 4.1	NS
Forward bend to the left	Pre-test	(-1.4) ± 5.8	(-9.8) ± 11.4	0.02
	Post-test	(-0.5) ± 6.0	(-10.4) ± 11.2	0.005
	Δ	0.9 ± 2.3	(-0.7) ± 4.3	0.19
Forward bend to the right	Pre-test	(-1.3) ± 4.9	(-10.4) ± 10.8	0.006
	Post-test	(-0.6) ± 6.0	(-10.5) ± 11.3	0.005
	Δ	0.7 ± 3.1	(-0.1) ± 3.6	NS
Dynamometry in LLL	Pre-test	284.6 ± 128.8	302.4 ± 115.3	NS
	Post-test	381.8 ± 152.1	314.3 ± 122.1	0.18
	Δ	96.8 ± 87.6	11.9 ± 93.6	0.013
Dynamometry in RLL	Pre-test	261.8 ± 115.2	313.2 ± 124.0	NS
	Post-test	356.8 ± 144.1	337.4 ± 140.1	NS
	Δ	94.9 ± 78.1	24.2 ± 108.1	0.043
Sit and stand up chair test	Pre-test	11.0 ± 2.5	11.6 ± 3.9	NS
	Post-test	11.9 ± 2.9	11.4 ± 2.6	NS
	Δ	0.9 ± 1.8	(-0.2) ± 3.1	*0.25*
2-minute step test	Pre-test	55.6 ± 9.0	52.4 ± 16.9	NS
	Post-test	57.5 ± 12.0	51.8 ± 15.7	NS
	Δ	1.9 ± 6.2	(-0.6) ± 8.4	NS
*IPAQ – high-intensity PA* *(Number of patients)*	*Pre-test*	*6* *28.2 ± 57.3 minutes/day*	*7* *53.3 ± 73 minutes/day*	*NS*
	*Post-test*	*12* *55.8 ± 98.8 minutes/day*	*8* *46.5 ± 158.2 minutes/day*	*0.03*
*IPAQ – moderate-intensity PA* *(Number of patients)*	*Pre-test*	*7* *118.3 ± 164.2 minutes/day*	*6* *117.9 ± 112.3 minutes/day*	*NS*
	*Post-test*	*4* *142.5 ± 137.3 minutes/day*	*7* *115.7 ± 96.1 minutes/day*	*0.06*
*IPAQ – low-intensity PA (Number of patients)*	*Pre-test*	*3* *Walking 88.2 ± 66.2 minutes/day* *Sitting 228.6 ± 118.6 minutes/day*	*3* *Walking 153.6 ± 175.8 minutes/day* *Sitting 242.3 ± 189.7 minutes/day*	*NS*
	*Post-test*	*0* *Walking 110 ± 53.8 minutes/day* *Sitting 280 ± 165 minutes/day*	*1* *Walking 148.6 ± 105.1 minutes/day* *Sitting 295.7 ± 134 minutes/day*	*NS*

n, number; NS, non-significant; Δ, delta (pre-test results minus post-test results); BMI, body mass index; WHR, waist-hip ratio; L, left; R, right; LLL, left lower limb; RLL, right lower limb; PA, physical activity.

We observed a partial yet significant improvement in flexibility of the larger joints (especially in the shoulders and forward bends) in group I compared to group C (p=0.005–0.012; **Table 6**). Based on dynamometric evaluation, muscle strength increased significantly in both lower limbs (left-lower limb; p=0.013, right-lower limb; p=0.043) in group I compared to group C (**Table 6**).

**Figure 3:**
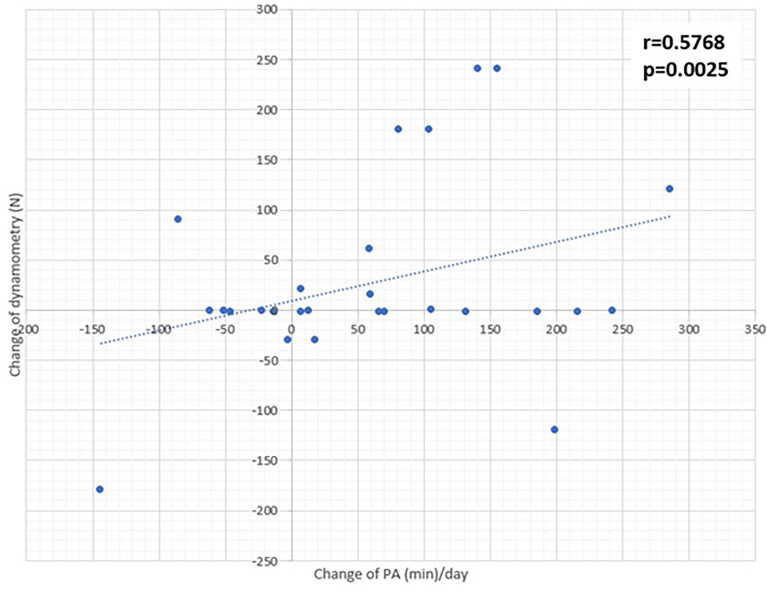
Correlation of dynamometry and PA changes detected after interventional program.

The structured exercise intervention had a positive trend on the improvement of fitness in group I compared to group C (p=0.25; **Table 6**). Additionally, there were positive correlations between high-intensity physical activity in minutes/day (according to IPAQ results) and flexibility parameters (r=0.47; p=0.007), fitness (r=0.453; p=0.011) and dynamometry (r=0.58; p<0.0025) (**Figure 3**).

## Discussion

Reduced PA is common in patients with DM (24), especially in those with DFS (25). However, DFS is preventable with structured PA (26). An increase in PA can be achieved either with a suitably chosen PA intervention (traditional exercise involving group training, aerobic exercise, weight training, etc.) or by increasing the intensity level of an existing PA programme (27, 28). Previous studies confirm that even short bursts of PA performed multiple times a day (walking up stairs or running to catch a bus) have a positive, cumulative effect on physical fitness and metabolism of each subject (29).

Patients with DFS are often forced to offload the lower limbs for long periods of time. This has the effect of rendering their inactive lifestyle even more sedentary and reducing PA to a minimum. In order to halt the progression of risk factors leading to DFS, structured PA or an individually designed exercise programme is essential (29). Any reduction in PA in diabetic patients decreases mobility and, with mechanical stimuli to the joints diminished, exacerbates LJM. The difference in LJM between diabetic patients and the healthy population is significant (30, 31). Unfortunately, the effect of reduced PA in patients with DFS extends to those with DFS in remission. Even in these patients, excessive stress on the soft tissues of the foot, which is often caused by varied PA, is not advised. Therefore, PA in this group of individuals should be increased gradually and preferably under medical supervision (32, 33). In any case, deconditioning will generate necessarily low-intensity PA. Because DFS patients typically exhibit lower tolerance in the soft tissues and skin of the feet to any kind of load, there is an increased dangerous risk of foot stress caused by intermittent and random increases in PA (34). Therefore, varied PA is not recommended. Armstrong et al. found that ulceration development is usually preceded by a 2-week increase in PA variability in the absence of any increase in average daily activity (32, 35). Informed by this and other studies, our intervention trial focused on increasing average daily activity as well as the total number of PA minutes to a minimum of 150 minutes per week (as recommended by WHO). The exercise programme was designed to last 12 weeks in the experimental group to ensure the lowest variability of PA, the gradual increase of the fitness load to avoid diabetic foot complication (mainly recurrences of diabetic ulcers). We have planned a study design to ensure the fitness program will have sufficient duration to influence the clinical impact on the evaluated outcomes. At the beginning of the study, IPAQ results for both groups were almost identical, with 18.8% of subjects in both groups reporting they engaged in low-level PA. In group I and group C, 43.7% and 37.5% of individuals, respectively, performed weekly PA at an intermediate level (min. 600 MET minutes/week), with 37.5% and 43.7% of subjects, respectively, performing high-level PA (min. 3000 MET minutes/week). However, the intervention resulted in a significant increase in PA levels in group I, with the number of individuals performing high-level PA increasing to 12 and low-level PA reducing to a minimum. In the control group, the results were almost identical to those obtained at the beginning of the study.

Despite the increase in IPAQ, we did not prove higher recurrence of DFU in the intervened group when compared with group C. The proportion of individuals in the control and experimental groups excluded due to RFU recurrence or other foot problems was the same (15.8%; 3/19). According to a study by Lemaster et al. (2008), which focused on achieving improvements in strength, balance and gait, low-intensity physical intervention reduced long-term skin lesions by more than 44%. Research indicates that this type of PA does not dramatically increase the risk of DFS recurrence, as also confirmed by our study. These positive results can be attributed to the overall beneficial effect on microcirculation and to the improvement of biomechanical parameters (36).

Our structured exercise programme led to an improvement in large joint flexibility, muscle strength and fitness. The intervention was specifically designed to be easy to implement so that participants would feel encouraged to improve their performance. The majority of patients in the intervened group reported subjective improvements in musculoskeletal function, mood as well as an increased desire to extend their exercise or walking time after the study. Following the 3-month intervention, more than 50% of individuals from group I contacted our physiotherapists to express their interest in maintaining or increasing their level of PA. …Since all study subjects were from all over the Czech Republic, it was not possible to train regularly with the patients and physiotherapist together. We arrange a special training lasting 30 – 60 minutes, when all exercises were demonstrated, until the patients performed the exercise correctly. The exercises were chosen to be easily completed and the individual did not have to be checked by physiotherapist. The training protocol implementation was regularly checked by fulfillment of training logs. Moreover, study physiotherapist was available on the phone 24 hours for 7 days a week. Interestingly, 10 out of 16 probands in the experimental group lost weight (an average of 1.8kg) compared to the control group.

SFT scores revealed improved shoulder flexibility and flexion in the intervention group compared to controls. Other SFT parameters did not differ significantly. SFT is rarely used in studies on DM (37, 38). Only one study has examined the impact of PA intervention on fitness in people with DM (39) SFT is not commonly used in recent studies. However, improvement in fitness, gait, balance and biomechanical abnormalities due to physical activity in diabetic patients has been widely studied in past (37–39), especially in patients with diabetic neuropathy (40–47). Thus, to our knowledge, the data documented in our study of patients with DFS in remission are unique. Considering the test battery takes as much as 30-40 minutes in all to perform, it is not entirely suitable for a group of elderly patients with serious comorbidities (14, 17, 48–51). Åström et al. found that the presence and severity of DM negatively affected the overall SFT score (52). Therefore, the battery of tests described above may be of limited use in a group of diabetic patients with comorbidities. But considering our participants were supervised by medical staff over the course of the entire examination, and that the consent of the examining physician to perform PA was obtained beforehand, the panel of SFT tests was considered appropriate and duly carried out. It should also be noted that the SFT is generally recommended as a suitable method for testing the effectiveness of exercise programmes across populations, including individuals with DM (15).

Previous studies have documented the positive effects of PA on plantar pressure. Typically, maximal plantar pressure reduces in the heel area, midfoot, lateral plantar area, forefoot and central plantar area, where functional changes in the musculature are the most probable causes (9). In view of these considerations, we set about devising a simple yet functional programme focused on improving muscle strength. Based on our data, dynamometry in the lower limbs significantly improved in the intervention group, while isometric muscle strength in both lower limbs was significantly higher in group I compared to group C.

Many of the studies that have investigated foot biomechanics in diabetic patients are similar in design (53–57). In agreement with our study, Francia et al. (2015) demonstrated the positive effect of PA intervention on ankle joint mobility, muscle strength and gait speed in patients with long-lasting diabetes. They compared a group of 26 diabetic patients (without differences in neuropathy) with 17 healthy respondents. Differences in ankle mobility between the healthy respondents and patients with diabetes were significant, with plantar flexion reducing by 36% and dorsal flexion by 23% in diabetic patients. With regard to muscle strength in diabetic patients, plantar flexion reduced by 51% and dorsal flexion by 30%. After 12 weeks of supervised exercise, joint mobility and muscle strength had significantly improved and walking speed had doubled. However, the diabetic cohort used in this study was small. In addition, their follow-up was not long enough to determine whether the intervened subjects had continued to exercise (or vice versa) or whether the 12-week exercise intervention had affected DFS development.

Similarly, a limitation of our study is the low numbers of enrolled individuals in contrast to previously promised count (ClinicalTrials.gov), as we had to terminate the study prematurely due to the Covid-19 pandemic and were unable to recruit additional patients with DFS in remission. Secondly, all exercise was performed at home without any direct follow-up by physiotherapists. Patients were informed about the exercise intervention in detail and supplied with both a training diary and training plan. However, the programme was voluntary and, as such, left to the discretion of the participants. Follow-up was carried out over the phone. Since probands lived in different parts of the country, it was not feasible for everyone to train under the supervision of a physiotherapist for the recommended 3 weekly sessions. Adherence to the intervention program was monitored by a training diary, which the I group had to fill in. From the beginning, they were strongly forced to fill in truthfully, and if the study subjects missed something, they should write it down (for example, illness or other ailments). Data from the training logs showed that the study subjects compliance was 90% in I group.

As demonstrated in this study, an age-appropriate structured intervention exercise programme can improve biomechanical parameters, such as flexibility of certain larger-joints, muscle strength and fitness in patients with DFS in remission without increasing the risk of recurrence. Given the potential to promote self-capacity, self-care and quality of life in these patients, incorporating such an intervention programme as part of daily podiatric clinical practice is strongly recommended (58). Effect of BIONEDIAN on the rest of biomechanical aspects (plantar pressures), quality of life and metabolic parameters will be further evaluated and discussed in next statistical analyses.

## Data Availability Statement

The original contributions presented in the study are included in the article/supplementary material. Further inquiries can be directed to the corresponding author.

## Ethics Statement

The studies involving human participants were reviewed and approved by FTN and IKEM’s ethics committee. The patients/participants provided their written informed consent to participate in this study.

## Author Contributions

EV, JH, RJ and VF contributed to the study protocol, researched the data, detailed and reviewed the manuscript. MD, VW, RB and AJ reviewed/edited the manuscript. KK and BP analyzed the data and VL performed the statistical analysis. All authors were equally entitled to query any aspect of the data, either directly or through independent analysis. All authors contributed to the article and approved the submitted version.

## Funding

This study was supported by GAUK 546417,NU20-01-00078 and the Ministry of Health of the Czech Republic through its Conceptual Development of Research Organisations programme (Institute for Clinical and Experimental Medicine – IKEM, IN 00023001).

## Conflict of Interest

The authors declare that the research was conducted in the absence of any commercial or financial relationships that could be construed as a potential conflict of interest.

## Publisher’s Note

All claims expressed in this article are solely those of the authors and do not necessarily represent those of their affiliated organizations, or those of the publisher, the editors and the reviewers. Any product that may be evaluated in this article, or claim that may be made by its manufacturer, is not guaranteed or endorsed by the publisher.
